# Object Detection Improves Tumour Segmentation in MR Images of Rare Brain Tumours

**DOI:** 10.3390/cancers13236113

**Published:** 2021-12-04

**Authors:** Hamza Chegraoui, Cathy Philippe, Volodia Dangouloff-Ros, Antoine Grigis, Raphael Calmon, Nathalie Boddaert, Frédérique Frouin, Jacques Grill, Vincent Frouin

**Affiliations:** 1 Université Paris-Saclay, Neurospin, CEA, 91191 Gif-sur-Yvette, France; cathy.philippe@cea.fr (C.P.); Antoine.GRIGIS@cea.fr (A.G.); 2 Pediatric Radiology Department, Hôpital Necker Enfants Malades, APHP, IMAGINE Institute, Inserm, Université de Paris, 75015 Paris, France; volodia.dangouloff-ros@aphp.fr (V.D.-R.); raphael@calmon.med.br (R.C.); nathalie.boddaert@aphp.fr (N.B.); 3 LITO U1288, Inserm-Institut Curie, 91400 Orsay, France; frederique.frouin@inserm.fr; 4 Department of Pediatric and Adolescent Oncology, Gustave Roussy, Inserm U981, Université Paris-Saclay, 94800 Villejuif, France; Jacques.GRILL@gustaveroussy.fr

**Keywords:** segmentation, object-detection, brain tumour, DIPG, deep learning, domain adaptation

## Abstract

**Simple Summary:**

This study evaluates the impact of adding an object detection framework into brain tumour segmentation models, especially when the models are applied to different domains. In recent years, multiple models have been successfully applied to brain tumour segmentation tasks. However, the performance and stability of these models have never been evaluated when the training and target domain differ. In this study, we identify object detection as a simpler problem that can be injected into a segmentation model as an a priori, and which can increase the performance of our models. We propose an automatic segmentation model that, without model retraining or adaptation, showed good results when applied to a rare brain tumour.

**Abstract:**

Tumour lesion segmentation is a key step to study and characterise cancer from MR neuroradiological images. Presently, numerous deep learning segmentation architectures have been shown to perform well on the specific tumour type they are trained on (e.g., glioblastoma in brain hemispheres). However, a high performing network heavily trained on a given tumour type may perform poorly on a rare tumour type for which no labelled cases allows training or transfer learning. Yet, because some visual similarities exist nevertheless between common and rare tumours, in the lesion and around it, one may split the problem into two steps: object detection and segmentation. For each step, trained networks on common lesions could be used on rare ones following a domain adaptation scheme without extra fine-tuning. This work proposes a resilient tumour lesion delineation strategy, based on the combination of established elementary networks that achieve detection and segmentation. Our strategy allowed us to achieve robust segmentation inference on a rare tumour located in an unseen tumour context region during training. As an example of a rare tumour, Diffuse Intrinsic Pontine Glioma (DIPG), we achieve an average dice score of 0.62 without further training or network architecture adaptation.

## 1. Introduction

Diffuse Intrinsic Pontine Glioma (DIPG) is a rare brain tumour located in the pons, mostly found in children between 5 and 7 years of age. It is considered one of the most aggressive paediatric tumours, with a survival rate of less than 10% beyond 2 years after diagnosis [1] and a median overall survival below 1 year [2]. The DIPG is categorised as a diffuse midline glioma, which is mostly characterised by a K27M mutation in genes coding for the histone H3 protein, and/or a loss of H3K27 trimethylation through EZHIP protein overexpression [3]. The location of the tumour and its corresponding genomic alteration makes the DIPG a completely different type of tumour from other High Grade Glioma (HGG) [4]. Thanks to a stereotactic biopsy that can be safely performed at diagnosis, molecular stratification correlated with survival [3] have been uncovered. However, due to the tumour location and its infiltrating characteristics, alternatives are being actively sought to find non-invasive biomarkers to propose innovative therapies and improve treatment monitoring.

The clinical management of DIPG patients includes MR neuroimaging at diagnosis and follow-up based on anatomical T1-weighted (T1w), Gadolinium-enhanced (T1Gd), T2-weighted (T2w) or Fluid Attenuated Inversion Recovery (FLAIR) images [5]. New imaging modalities, such as perfusion imaging, are thought to show pertinent indicators of the disease progression [6]. Parallel to this, new classification or prediction approaches based on image features, such as radiomics, allow MRI scans to be used for both disease stratification at diagnosis and progression monitoring [7]. Yet, learning from images classically requires large cohorts for which tumours are finely delineated. The rarity of DIPG added to the fact that segmentation is not part of the clinical routine procedure, making it difficult to obtain robust statistical classifiers or predictors. Automatic tumour segmentation based on transfer learning could theoretically alleviate this problem, circumventing the small number of manual delineations which prevent directly and efficiently training these segmentation models.

**Figure 1:**
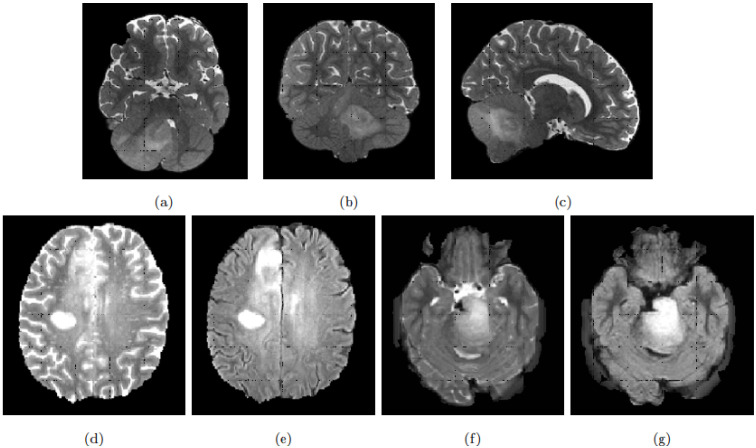
Top row: Example of T2w MRI scan of a patient with DIPG tumour extending beyond the pons. (**a**) Axial, (**b**) Coronal and (**c**) Sagittal slices. Bottom row: Example of glioblastoma and DIPG MRI scans, in axial slices. (**d**) glioblastoma T2w, (**e**) glioblastoma FLAIR, (**f**) DIPG T2w and (**g**) DIPG FLAIR.

In general, DIPG tumours have a central location involving more than 50% of the pons [8]. On MRI scans and due to its infiltrating nature, the tumour appears as an intrinsic expansion of the brainstem and not as a distinct foreign mass compressing the pons. However, the tumour is not always restricted to the pons and it can infiltrate other compartments of the central nervous system such as the cerebral peduncles and supratentorial midline or the cerebellum [9]. The deformation of the pons induced by the tumour and its infiltrating nature makes its detection and delineation non-trivial. On the T2w scans, the tumour presents a hyper-intense signal while it appears hypo-intense with indistinct tumour margins on T1w scans. Enhancement following gadolinium injection (T1Gd) is inconstant and often absent. Finally, the tumour is relatively homogeneous on FLAIR modality [8]. Figure 1 exhibits a DIPG tumour on different modalities.

DIPG shares some of its visual characteristics with the glioblastoma, especially on T2w and FLAIR modalities. However, glioblastoma presentation differs on T1w and T1Gd, with the absence of a necrosis component in the DIPG, and the gadolinium enhancement which is more intense and always present for the glioblastoma [10]. Our aim is to exploit the existing macroscopic visual similarities of DIPG with glioblastoma or low-grade gliomas, to train a two-step robust model able to infer DIPG segmentations.

Because rare tumours present some visual similarities with common tumours, in the lesion and around it, one may split the tumour delineation problem into two steps: object detection and segmentation. For each step, trained networks on common lesions could be used on rare ones following a domain adaptation scheme without extra fine-tuning. Our work suggests different architectures to solve the segmentation and detection tasks and two combination strategies of the two tasks. We assessed the performance of our strategies in three different configurations: Using the same tumour as in the training, using a different tumour located in the supratentorial region as in the training and finally using a different rare tumour located in the brainstem, an unseen region during the training.

In the section Related Works, we present current work involving brain tumour segmentation and show their limitation concerning the problem of rare tumours. Then, the section Material and Methods describes our models for each of the two tasks, detection and segmentation, and the proposed combination strategies. Our strategies are trained on a large general database available in neuro-oncology, namely the BRaTS database [11,12,13]. In the section Experiments and Results, we use this publicly available database (subdivided in one train and two test sets) to obtain several global segmentation models and to assess them in the task of providing segmentations on the High Grade Gliomas and Low Grade Gliomas. These data are also used to analyse the relationship between detection and segmentation performance. Finally, results of inference in new DIPG images are presented and discussed.

## 2. Related Works

Numerous studies have successfully used MRI scans for profiling in many adult tumours. In the case of DIPG, MRI profiling potential has been assessed only in very recent works, as in the work of Leach et al. [14] where radiological descriptors, among others, have been used to study survival. However, they did not make use of tumour segmentation, but rather visual assessments achieved by a team of neurologists. Accurate tumour volume estimation protocols have been proposed by Singh et al. [15] for the DIPG. However, they studied only eight patients for whom a neuro-radiologist executed a manual delineation to obtain the volume estimates. This is not scalable to a cohort of more than a hundred patients, and it raises concerns about the stability and bias of the results because of inter-operator variability. To our knowledge, no automatic segmentation strategy has been proposed yet for DIPG.

Segmentation approaches based on the region competition driven by multivariate statistical distribution analysis of the image grey levels [16] could be applied. Indeed, in the case of DIPG, the location of the tumour is broadly known and the limiting initialising step of the region competition methods should be easy. Yet, it is still to be solved as the DIPG tumour, with its specific infiltrating pattern, has an impact on various brain structures and presents highly variable appearances in the different image modalities. Consequently, these methods, which remain highly dependent on initialising seed, would have to be trained heavily. Moreover, these approaches are very sensitive to the availability of all the imaging modalities present during the learning of the model. They would require a database of expertly labelled images of the size of BraTS (around 250 samples).

Deep learning techniques have shown great success in a wide variety of tasks. Convolution Neural Networks (CNN) [17], in particular, have proven their efficiency in computer vision tasks such as classification, object detection and segmentation, as they have shown a great capacity to extract highly relevant features for the tasks at hand. These CNN have been successfully used on natural images and medical scans. Numerous works have been proposed for the automatic segmentation of cerebral tumours, notably for glioblastomas. In general, these techniques use either patch-based segmentation or end-to-end segmentation.

Patch-based segmentation relies on multiple patches from the same image for the full segmentation under the assumption that the central region of similar patches will have similar labels. With these techniques, multiple segmentations, extracted from different neighbouring patches, can be proposed for the same set of voxels, and thus increase the stability of these models. Havaei et al. [18] and Kamnitsas et al. [19] are among several works related to patch-based techniques. While the first segments the 3D volumes using 2D patches fed into a simple multi-path network, the latter uses 3D patches given into a more complex model. However, these models require more computational resources compared to end-to-end models and introduce a sampling problem, knowing that a brain tumour occupies on average 7% of the brain volume in the studied datasets.

End-to-end segmentation predicts labels from the whole volumes or slices. Contrary to patch-based segmentation, here the model scope is not restricted. Thus, the model has more information to work with, but also it has to determine the tumour location. Most recent studies have found great success using encoder-decoder architectures. Myronenko A. et al. [20] won the BraTS’18 challenge with a segmentation network that uses 3D MRI scans and employs multitasking to help compensate for the limited dataset size. Meanwhile, Isensee et al. [21] obtained excellent results, ranking 2nd on BRaTS’18, using the classic UNet architecture with minimal modifications. More recent works in brain tumour segmentation focus on UNet and its iterations [22,23,24] and exhibit the power of this architecture to solve the segmentation problem.

Segmentation models can be improved by providing priors. Since cerebral tumours can come in different shapes [25] and locations [26], we cannot use this information as priors, as proposed in numerous works. Bounding-boxes around target objects can be used instead. Lempitsky et al. [27] incorporated user-provided bounding-boxes as a way to add topology and shape information to their loss function and applied it to the natural images object segmentation problem. Rosana et al. [28] proposed to feed their UNet architecture with their user-provided bounding-box masks in parallel to their input images. These propositions do not discuss the origin of the bounding-boxes, and while user-provided bounding-boxes can be reliable, automatically detected ones can introduce multiple issues, which we propose to study.

Segmentation approaches can work on 2D or 3D images. Using 2D images limits the scope available to the model and can result in discontinuities. 3D models might seem like a better solution, but these architectures are much more complex and have fewer training examples, making them hard to train. Some works propose hybrid models, which use both 2D and 3D inputs, such as Mlynarski et al. [29], who propose a neural network that uses 3D images combined with features extracted from different 2D images.

The approach we propose in this work combines object-detection and segmentation models, each trained independently. Thus, our method differs from the Mask R-CNN (Region-based CNN) strategy [30]. Indeed, Mask R-CNN has linked detection and segmentation architectures, which are trained concurrently, while our proposition relies on multiple different networks that do not share parameters.

In the context of tumour segmentation, our approach is among the methods that use bounding boxes as *a priori* to help segmentation task. Unlike BB-UNET, our approach suggests a way to obtain the bounding boxes and study their impact on the segmentation results. Solving the segmentation and detection tasks using separate networks allows us to reuse networks in a completely uncoupled way-which differs from the Mask-CNN. Using two architectures independently allows us to extract different features for each task which increases flexibility and robustness of our approach (the ensembling using the separate outcomes may bring a solution).

## 3. Material and Methods

### 3.1. Description of Datasets

Different datasets to train and test the models were used to benchmark our strategy. First we used the public dataset BraTS’19 [11,12,13] (last access January 2020), which comprises 254 patients diagnosed for High Grade Gliomas and 76 patients with Low Grade Glioma. These sets do not include any patient presenting a midline tumour and do not contain any patient presenting a DIPG. For each individual, 4 MRI volumes corresponding to T1w, T1Gd, T2w and FLAIR were available. These volumes were acquired with different clinical protocols, and with various scanners from multiple institutions (n=19) and originated from different studies.

From the HGG set, we isolated the 97 patients diagnosed with Glioblastoma Multifome (GBM) and belonging to the TCGA-GBM sub-cohort. From now on the HGG dataset refers to the 157 patients from BRaTs’19 HGG deprived of TCGA-GBM, and the LGG dataset refers to the 76 BRaTs’19 LGG patients. The HGG dataset will be divided into HGG${}^{train}$ and HGG${}^{val}$ and used for the training and validation sets, while the TCGA-GBM${}^{test}$ and LGG${}^{test}$ are used for testing our models. LGG${}^{test}$ comprises tumours different from the ones in the training but located in the same tissue (e.g., white matter in cerebral hemispheres).

The DIPG${}^{test}$ is a private dataset of 30 patients diagnosed with DIPG across 71 sessions. Informed consent of the use of clinical and radiological data was obtained and the protocol was approved by the local ethics committee of the reference institution. All the cases have the T2w and FLAIR modalities available. Manual delineation of the whole tumour was achieved for all test cases and checked on the most relevant modality (for some patients, ASL and diffusion MR images were used) [31,32]. These segmentations were obtained on images with a 4 mm${}^{3}$ resolution. DIPG${}^{test}$ comprises a tumour type different from the ones in the training phase and not located in the same tissue.

We are only interested in differentiation between tumoral and non-tumoral tissue, without determining the substructure of the tumour. The segmented compartments available in BRaTS’19 comprise the gadolinium-enhancing tumour (ET), the peritumoral edema (ED), and the necrotic and non-enhancing tumour core (NCR). For this study, we retained a binary tumour mask per patient defined as the union of all three compartment types. The segmentation information available in the subset DIPG${}^{test}$ corresponds originally to a binary tumour mask encompassing all the compartments.

### 3.2. Image Pre-Processing

All MRI scans received a two-step pre-processing. The first step corresponds to the data standardisation described in BraTS’19 [11]. The images were re-oriented to left-posterior-superior (LPS) coordinate system, co-registered rigidly into their respective T1Gd volume, interpolated to the 1 mm${}^{3}$ isotropic resolution and skull-stripped. The standardised images of HGG${}^{train}$, LGG${}^{test}$ and TCGA-GBM${}^{test}$ were obtained directly from BRaTS’19 site where they are available. The DIPG${}^{test}$ images were standardised by our group following the same protocol and using the open-source software FMRIB Software Library (FSL) [33]. The second step of the pre-processing was performed to harmonise the values obtained with a min-max intensity normalisation. Our choice of normalisation is justified by our intent to enhance tumour intensities, thus making our detection and segmentation tasks easier. The images were min-max normalised using the 5 and 95% percentiles in order to discard outliers, and out-of-range values were capped as in Equation (1) where *v* and $\hat{v}$ are, respectively, the original, and normalised grey level of a generic voxel of the image v:

(1) $$\hat{v}=\max\left(\min\left(\frac{v-percentile(v,0.05)}{percentile(v,0.95)-percentile(v,0.05)},1\right),0\right)$$

### 3.3. Detection-Segmentation Combination Strategy

#### 3.3.1. Input Data

We decided to train all our models on 2D slices as we can extract a greater number of different training examples from each 3D volume. Furthermore, we trained all our models only on axial slices since these slices have the best resolution in all the studied cohorts.

In this work, we only used the T2w and the FLAIR modalities. Our choice is justified by several considerations. First, HGG and DIPG MRI scans present similar local tumour patterns mostly in these two sequences. Furthermore, we are only interested in binary segmentation, and the modalities which best reveal all the different compartments of the tumour are the T2w and the FLAIR. Finally, the DIPG${}^{test}$ is representative of DIPG data obtained in the clinical context, which contains many subjects with missing sequences, but most subjects have either the T2w or the FLAIR available.

#### 3.3.2. Combination Strategies

To obtain a robust segmentation of brain tumours, we combined proven object-detection models and segmentation models. Considering we could not directly learn from a few labelled DIPG examples, we decided to train our models with HGG${}^{train}$ examples. The HGG and DIPG tumours present both similarities and differences (see Figure 1). Tumour intensities have comparable characteristics, while the ages of the patients, tumour locations and image qualities differ. We hypothesised that in a restrained zone around the tumour, HGG and DIPG present enough visual similarities to allow the training of a segmentation model from the sole HGG data and which would be able to segment reliably both types of tumours. We used an object detection model to define these restrained zones around the tumour and bypass the dissimilarities between the two cohorts.

We chose You Only Look Once (YOLO) [34,35] as our object-detection framework. For the segmentation, we benchmarked UNet [36] and Bounding-Box UNet (BB-UNet) [28]. It must be noted that both UNet and BB-UNet receive the whole 2D images as input for the training and inference; additionally, BB-UNet receives also an a priori bounding-box used internally to (non-exclusively) focus the learning segmentation process. Consequently, we examined two different procedures to combine the object-detection and segmentation. We called our first procedure Parallel YOLO UNet (pYU). In pYU, both YOLO and UNet are trained independently. In the inference phase, YOLO-generated bounding-boxes are merely used to mask UNet predictions, thus eliminating all segmented voxels outside the bounding-box. In our second approach, called Sequential YOLO BB-UNet (sYBBU), YOLO and BB-UNet are trained independently, but during inference, YOLO-generated bounding-boxes are provided as additional input to BB-UNet. As with pYU, we also used the bounding-boxes to mask the segmentation output.

#### 3.3.3. Final Masking

Let SEG, BBOX and GT be the sets of voxels belonging to the predicted segmentation, predicted bounding-box and ground truth, respectively. We define precision and recall for a set of voxels *M*, that in our context refers to the predicted segmentation mask or the predicted bounding-box mask, as:

(2) $$\begin{array}{cccc} Recall(M) & =\frac{\left|M\cap GT\right|}{\left|GT\right|} & Precision(M) & =\frac{\left|M\cap GT\right|}{\left|M\right|} \end{array}$$

The tumours we are interested in are all in a continous compact region. Thus, our approaches introduce a masking phase. Masking (versus no masking) will affect precision/recall scores in an anticipated direction, if we make an assumption that will be checked in our results: let *v* be a voxel and *P* a probability:

(3) P(v∈GT|v∈(SEG∩BBOX))≥P(v∈GT|v∈(SEG\BBOX))

Under this assumption, it is more likely to find a true positive inside the bounding-box than outside. Thus, it follows that:

(4) $$\begin{array}{c} Precision(SEG\cap BBOX)\geq Precision(SEG)\\ Recall(SEG\cap BBOX)\leq Recall(SEG) \end{array}$$

Masking will always result in a decrease in the recall (amount of the tumour detected). However, we assume that the decrease in the recall will be outweighed by the increase in precision.

#### 3.3.4. Ensembling the Inferences

Each model is trained two times on mono-modality, one with the T2w and another time with the FLAIR. This makes our approach resilient to missing data. Depending on the data at hand, we retain the inference obtained from the single modality available or we combine the two inferences. In this latter case, we merged the predictions using a weighted average. As described by [37], we propose to find the optimal weights according to the optimisation problem, where *N* is the number of validation cases, ${\hat{y}}_{v}^{FLAIR}$ (resp. ${\hat{y}}_{v}^{T2w}$) is the models confidence scores for the FLAIR (resp. T2w) on a voxel, $label({\hat{y}}_{v})$ is the thresholded confidence score and takes the values 0 (for non-tumoral voxels) or 1 (for tumoral voxels), $\omega^{*}$ the optimal weight:

(5) $$\begin{array}{c} \mathrm{with}\;{\hat{y}}_{v}\left(\omega \right)=\omega \times {\hat{y}}_{v}^{FLAIR}+\left(1-\omega \right)\times {\hat{y}}_{v}^{T2w}\\ \omega^{*}=\underset{\omega }{argmin}\left(-\frac{1}{N}\sum_{k=1}^{N}\left(\sum_{v\in DISCR}GT_{v}\times \log\left(\hat{y_{v}}\left(\omega \right)\right)+\left(1-GT_{v}\right)\times \log\left(1-\hat{y_{v}}\left(\omega \right)\right)\right)\right)\\ s.t.0\leq \omega \leq 1 \end{array}$$

To find the optimal weight, we used the HGG${}^{val}$ set. To better fit our needs, we adapted the optimisation problem by only considering the $DISCR=\{v|label\left({\hat{y}}_{v}^{FLAIR}\right)\neq label\left({\hat{y}}_{v}^{T2w}\right)\}$ (i.e., the set of voxels where T2w output label and FLAIR output label are different). This is justified by the fact that the weighted average does not change the predicted label if both models predict the same label.

Ensembling was used both after the detection inferences and after the segmentation inferences. More precisely, we used the confidence scores of the YOLO models to compute an ensembled bounding-box prediction, and the UNet and BB-UNet scores for an ensemble segmentation prediction. For the segmentation, the ensembling phase is prior to the bounding-box masking.

We used Brent’s method [38] to solve the optimisation problem (Equation (5)) and SciPy’s optimisation package [39].

**Figure 2:**
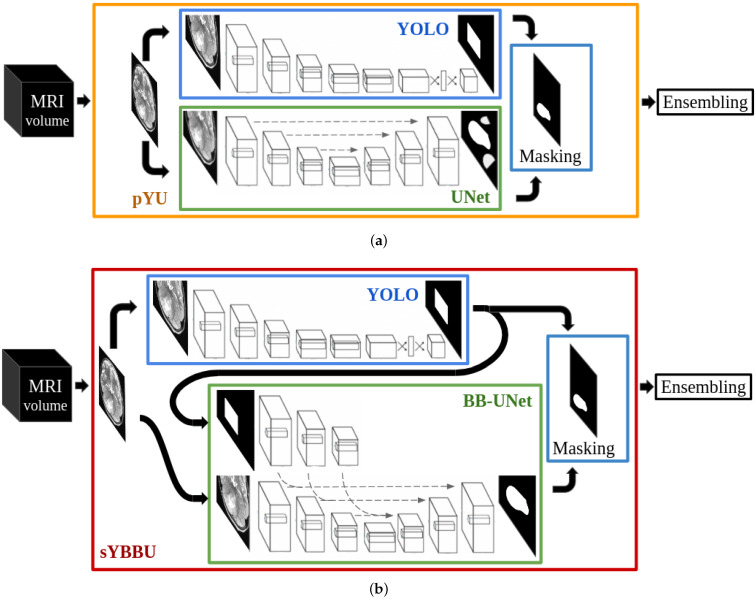
The two approaches pYU and sYBBU. (**a**) Parallel YOLO UNet (pYU) model; (**b**) Sequential YOLO BB-UNet (sYBBU) model.

Figure 2 illustrates the two approaches, pYU and sYBBU, sketched in their inference stage. Supplementary Figure S1 and Figure S2 show the different training and inference possibilities and combinations.

### 3.4. Reusing Off-the-Shelf Networks

#### 3.4.1. You Only Look Once (YOLO)

You Only Look Once (YOLO) framework is a multi-scale object detection neural network. YOLO is designed to detect multiple objects of different classes on natural images. The input image is divided into an S×S grid of cells. The cell where the centre of the object falls into is responsible for predicting the bounding-box and class of the objects. Each cell predicts *B* bounding-boxes, confidence related to the existence of an object in each bounding-box, and conditional class probabilities related to the object instances.

We used the YOLOv5, implemented by Ultralytics [40]. This network was pre-trained on 416×416 images from the Common Objects In Context (COCO) dataset [41]. We used transfer learning to fine-tune the model parameters for the tumour detection task. We resized our 250×250 input images to the 416×416 dimension using zero padding. The model was trained to detect the smallest bounding-box around the tumour. Since YOLO is pre-trained on RGB images, we transformed our grey-scale images into RGB images by copying our input image into the three channels.

For the hyper-parameter *B*, we kept its default value 3. Because YOLO makes predictions with a multi-scale approach, *S* took successively 3 values in which the prediction is made. For 416×416 input images, the values taken were 13, 26, 52. Furthermore, we were interested in detecting only one object, namely the tumour (using the –config file requested by the software).

Finally, starting from the pre-trained model, we fine-tuned it for 100 epochs, using an initial learning rate of 0.001. Other default training parameters were kept. Main parameters are listed in the Supplementary Table S1.

We assume that tumours are 3D-connected-component volumes. However, as the detection model took axial 2D images as input, there was thus no guarantee to obtain a connected component object in a plane perpendicular to the axial plane. The model might miss the tumour on some slices of the volume, or detect tumours on isolated slices. To overcome this issue, we used a morphological closing of the bounding-boxes, followed by an opening, along the perpendicular axis to the axial plane, with a kernel size of (1,1,6) voxels.

#### 3.4.2. UNet and BB-UNet Models

For the segmentation step of the tumours, we studied two models. First, we used UNet, a fully convolutional neural network, which is classically used for biomedical image segmentation [36]. Similar to an auto-encoder, it has two paths: an encoding path consisting of the stacking of convolutions, non-linear activations and max-pooling; and a decoding path which consists of convolutions, non-linear activations and transposed convolutions. Skip connections are used between each encoding layer and its symmetric decoding layer. Our network is 5 levels deep on each path. We used the rectified linear unit (*ReLU*), defined as ReLu(u)=max(u,0), as the non-linear activation function.

Second, we used the BB-UNet [28] model, whose architecture is similar to that of the UNet, except that it takes a binary bounding-box mask as additional input. We added to the UNet a bounding-box path parallel to the encoder path. The binary mask follows similar transformations to the main image. At each skip connection, we carry out an element-wise multiplication between the encoded image and encoded bounding-box. The role of these bounding-boxes is to discourage the network to look beyond it. Our BB-UNet models were trained using the ground truth bounding-boxes obtained as the smallest bounding-boxes comprising all the tumour mask (in 2D). As stated before, we used the YOLO-predicted bounding-box during the inference phase.

For both models, the last layer has a soft-max activation, and we used a binary cross-entropy as a loss function, defined as:

(6) $$Loss\left(\hat{y},y\right)=-\hat{y}\cdot \log\hat{y}+\left(1-y\right)\cdot \log\left(1-\hat{y}\right)$$

where **y** and $\hat{y}$ are the ground-truth labels and the network confidence score matrices, respectively.

We implemented both UNet and BB-UNet using Pytorch [42]. The neural networks were trained on 250×250 grey-scale images for 100 epochs, with an initial learning rate of 0.001 and the Adam optimiser [43]. Given the size of the used dataset (≥9000), no data augmentation was used.

#### 3.4.3. Deepmedic

We compare all our results to a reference, patch-based brain lesion segmentation network, namely Deepmedic developed by Kamnitsas et al. [19]. Deepmedic is an 11-layers deep, double-pathway, multi-scale, 3D CNN. Deepmedic achieved state-of-the-art results on brain tumour segmentation on BraTS’15, and it is continuously updated. We trained Deepmedic on mono-channel twice, with T2w and FLAIR, to make the results comparable with our approaches. Contrary to our models, input images for Deepmedic were normalised using a z-score to remain in line with the network procedure.

We used the implementation from https://github.com/deepmedic/deepmedic (accessed on 3 December 2020). We kept the default values for the hyper parameters as proposed by the original paper, including the number of layers, filters, learning rate, and optimiser.

## 4. Experiments and Results

### 4.1. Experimental Designs

To conduct our experiments, we divided the HGG dataset into a 90% training set and 10% validation set. We tested all the models on the TCGA-GBM${}^{test}$ dataset, the LGG${}^{test}$ dataset and 30 patients (71 sessions) of DIPG${}^{test}$. Table 1 sums up the dataset sizes.

To assess the performance of our approaches on the different test datasets, we used the provided segmentation labels to compute precision and recall (see Equation (2)), alongside the Dice index (Equation (7)), with *M* the predicted binary mask, and GT the binary ground-truth. These metrics were measured after the 3D reconstruction of the binary masks. We note that the object-detection outputs are also binary masks.

(7) $$\begin{array}{cc} Dice(M) & =\frac{2\times |M\cap GT|}{\left|M|+|GT\right|} \end{array}$$

On the TCGA-GBM${}^{test}$ dataset, we performed a correlation analysis between the ensembled bounding-box performance and the ensembled segmentation performance, in order to establish the impact of the object-detection step on the final segmentation. Furthermore, since BB-UNet models were trained with the ground-truth bounding-boxes while inference was performed using YOLO predicted bounding-boxes, we analysed the impact of the used bounding-boxes on the prediction performance of the networks.

On the DIPG${}^{test}$ dataset, we compared the object-detection performance with a generic bounding-box around the pons. This bounding-box was manually extracted from a template [44] with an enlargement of approximately 50% on each side. Supplementary Figure S3 summarises the experimental design chosen to evaluate the methods.

### 4.2. Benchmark Results

#### 4.2.1. Object-Detection Results

Table 2 and Table 3 give the results of the detection phase on the TCGA-GBM${}^{test}$ and LGG${}^{test}$ datasets. Overall, both the FLAIR and the T2w obtain a very high recall and a relatively low precision score. The merging of both modalities helps further improve the recall and the stability of the predictions (lowering the standard deviations) while lowering the precision. Low precision scores were expected in this phase since the predictions are piece-wise squares while tumour shapes are complex meshes. Therefore, the precision score depends heavily on the tumour shape and orientation. One must also note that a tumour generally occupies around 7% of the brain, in the studied dataset, which impacts the precision score. To choose the best model, it is important to remember that the main objective of this phase is to generate priors for a segmentation. It is therefore imperative to reliably detect the whole tumour (implying high recall), even if it comes with lower precision.

The detection framework achieved better performance in the TCGA-GBM${}^{test}$ dataset than in LGG${}^{test}$. This was expected since our model was solely trained to detect high-grade-gliomas. Even if the performance was degraded for the LGG${}^{test}$ dataset, this decrease is moderate, especially when comparing the results of the ensembled model. This shows that the object-detection model is able to detect different types of tumours that occur in the same tissues of the brain.

Morphological opening and closing showed a minimal effect. However, as these effects were always positive on both precision and recall, we kept them in our detection process.

#### 4.2.2. Segmentation Results

YOLO bounding-boxes, obtained in the previous phase, were used during the segmentation. Each segmentation model uses bounding-boxes obtained from the same input image and modality. The bounding-boxes used for the segmentation are all post-processed by the morphological transformations.

##### Segmentation Results on TCGA-GBM${}^{test}$

Table 4 describes the results obtained for the segmentation of TCGA-GBM${}^{test}$. A voxel is considered tumoral if its confidence score ${\hat{y}}_{v}$ is above 0.5. As expected, precision scores were considerably higher than during the detection phase, however, this came with a decrease in recall.

The UNet models performed poorly on TCGA-GBM${}^{test}$ compared to the other models. Indeed the mean Dice index ranged from 0.70 to 0.78, which is below the other models’ averages, with values greater than 0.84. This was mainly due to their low precision scores. A deeper look into the results showed that UNet segments healthy bright spots of the brain the same way it segments the bright spots indicating the presence of the tumour, especially edema. On the other side, UNet gave comparable results in the recall metric (i.e., percentage of the tumour detected).

Moreover, we can see a clear improvement after UNet segmentations were masked with the predicted bounding-boxes, i.e., the pYU model. Mean precision scores increased by nearly 15% in all configurations, while the standard deviations were reduced by nearly half. These results suggest that most of the false positives are outside of the bounding-boxes, which is in line with the assumption stated by Equation (3). These improvements came with a slight decrease in the recall, of around 1%. We consider this decrease is minor compared to the benefits of masking with bounding-boxes in precision.

**Figure 3:**
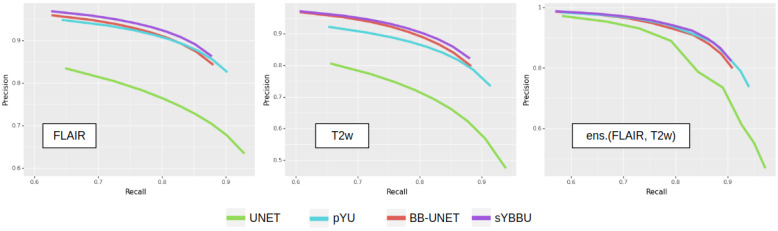
Mean precision-recall graphs of the different proposed segmentations on the TCGA-GBM${}^{test}$ dataset. To focus on the most interesting part of the plot, we only plotted the precision-recall scores for thresholds between 0.1 and 0.9. From the left to the right, Using the FLAIR, using the T2w, and ens.(FLAIR, T2w).

Furthermore, bounding-boxes also have a positive effect when they are used as inputs in the BB-UNet models. There is an increase in mean precision and a decrease in standard deviations of precision and Dice index. After masking, the results of UNet and BB-UNet are very similar, with BB-UNet coming slightly ahead, with an improvement in precision between 1% and 3%, and a recall that remained similar among all the models. Figure 3 exhibits clearly that models using the bounding-boxes perform better, especially sYBBU models. To compare the sYBBU model as the best approach using bounding-box with UNet that ignores them, we computed the AUC of the mean precision-recall graph. We obtained 0.91, 0.90 and 0.93 on the FLAIR, T2w and ens.(FLAIR, T2w), respectively, on the sYBBU model. Meanwhile, on the UNet model, we obtained 0.80, 0.83 and 0.90 using the FLAIR, T2w and ens.(FLAIR, T2w), respectively.

The precisions and recalls in segmentation using the bounding-boxes are strongly correlated with the respective precisions and recalls of the bounding-boxes detection, by a correlation score between 0.6 and 0.8 (see Supplementary Table S2 and Table S3). Of note, UNet results are also positively correlated with the object detection results (correlation between the precisions is 0.40 and correlation between the recalls is 0.66), though not as strongly as in the other models. This suggests that part of the performance is related to the images themselves, and some tumours are especially hard or easy to detect or segment for any model due to image quality or tumour visual characteristics. However, the overall performance is strongly dependent on YOLO’s ability to detect the whole tumour. This is shown on the Dice metric, which indicates a strong correlation between bounding-box recall and the Dice of models using the bounding-boxes, ranging from 0.61 to 0.72. This reinforces the strategy consisting in promoting recall over precision during the detection phase in order to obtain overall high performance.

Overall, FLAIR-based models perform better than T2w-based models. It appears that the FLAIR may reflect the diffuse characteristics of the tumour better, while in the T2w images, the intensity distribution of voxels inside the tumour is not as distinguishable from other bright regions of the brain. However, the ensembled models always perform better, across all configurations, and have equal or lower standard deviations. When computing the optimal weights to merge the models, we found ω=0.77 for pYU and ω=0.50 for sYBBU. However, the gain of an optimized weighted average, as opposed to a basic average, was below 1%. The weighted average improves the log-likelihood, but with little impact on the accuracy after binarization.

Table 5 shows the differences in metrics when ground-truth bounding-boxes were used for the FLAIR in BB-UNet. BB-UNet with ground-truth bounding-boxes was unable to detect, on average, 10% of the tumour. When YOLO bounding-boxes were used, a 6% decrease in recall was observed. This exhibits that two-thirds of the missed voxels are inherently related to BB-UNet and not to errors in YOLO bounding-boxes. Given these results, we can say that YOLO bounding-boxes are not the prevailing source of errors and they are sufficient to be integrated into our detection-segmentation approach.

Concerning Deepmedic architecture, results on the FLAIR modality are slightly below results obtained with the proposed approaches. The Deepmedic model seems to prioritise high precision over recall. However, Deepmedic trained with the T2w failed to give any meaningful result, with an average Dice index of 0.08, which makes the T2w unusable in an ensembled model.

##### Segmentation Results on LGG${}^{test}$

Table 6 shows the segmentation results obtained on the LGG${}^{test}$ dataset. Overall, the proposed models exhibit comparable results with those obtained on the TCGA-GBM${}^{test}$ dataset but show an average drop in the Dice metric of 0.05. This reduction was expected since the networks were solely trained on High Grade Gliomas and were not readapted for the Low Grade Glioma cases. Unlike the proposed models using bounding-boxes, UNet showed poor performance on the LGG${}^{test}$ dataset. However, the pYU model shows an improvement in the overall results by increasing precision by 30% at the cost of a mean decrease of 10% of the recall. We obtained an AUC score of 0.80, 0.87 and 0.89 for the FLAIR, T2w and ens.(FLAIR, T2w), respectively, when using the sYBBU. Comparatively, we obtained an AUC score of 0.71, 0.73, 0.80 for the FLAIR, T2w and ens.(FLAIR, T2w), respectively, when using UNet only. See also Supplementary Figure S4.

The proposed procedures outperformed the Deepmedic network. On average, the Dice metric was between 6% and 9% lower for Deepmedic compared to our models. This exhibits the robustness of our strategy. Similar to the TCGA-GBM${}^{test}$ dataset, the Deepmedic model seems to prioritize high precision (the highest on all the models) over the recall.

### 4.3. Segmentation Results on DIPG${}^{test}$

From the 30 DIPG patients, 71 sessions were available obtained at different follow-up visits. Table 7 shows the inference detection results obtained on 62 out of the 71 DIPG${}^{test}$ sessions. The detection step failed to identify the tumour region (tiny bounding-box with recall <0.015) in 9 sessions (13% of the sample). Supplementary Table S4 shows detection results obtained on all 71 test sessions. Overall, the FLAIR exhibited significantly better results than the T2w, especially for the recall. Bounding-boxes obtained from the FLAIR show robust results. On average, 66% of the tumour is captured, with a mean precision equal to 61%. While the T2w alone failed to give significant results, ensembling T2w bounding-boxes with FLAIR bounding-boxes improves the recall by 7% while lowering its precision by 17% on average. Both FLAIR and ens. (FLAIR, T2w) bounding-boxes performed better than the generic bounding-box around the pons, which gives a 63% recall with a 20% precision. This shows that, even if the location of the tumour is known beforehand, the problem remains non-trivial because of the infiltrating nature of the tumour and its tendency to deform the surrounding tissue or structures (cerebellum, spinal cord, thalamus). Careful inspection of the nine cases with detection step failure indicates that the failure is mostly related to the tumours not being visible on the FLAIR and T2w MRI scans. Due to the low performance of object detection on T2w, we only used FLAIR and ens.(FLAIR, T2w) detection masks in the segmentation phase.

**Figure 4:**
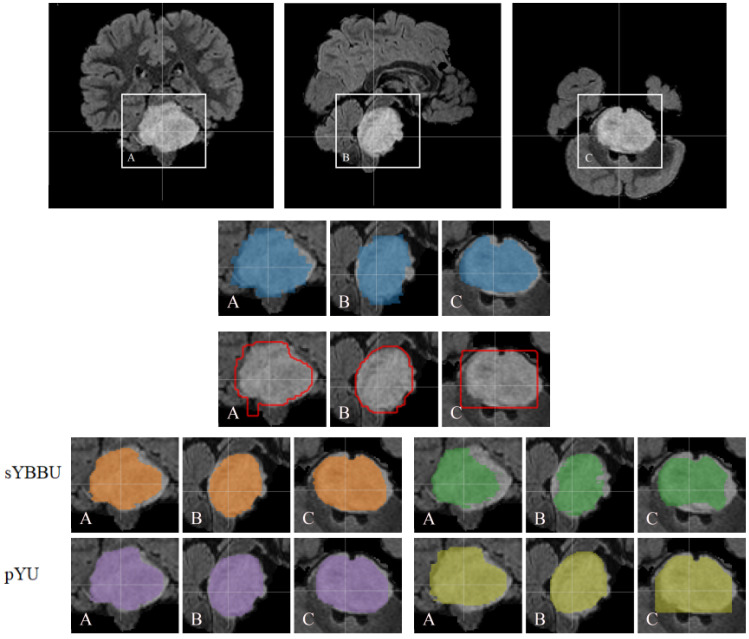
Segmentation results obtained on one case of DIPG are superimposed on the FLAIR background. The top row displays the complete patient images. Ground truth mask in blue. Yolo detection contours in red. sYBBU Segmentation with FLAIR in orange. sYBBU Segmentation with T2w in green. pYU Segmentation with FLAIR in purple. pYU Segmentation with T2w in yellow. A: coronal, B: sagittal and C: axial.

Table 8 shows pYU and sYBBU segmentation results using FLAIR detection masks. Supplementary Table S5 shows segmentation results with the combined ens.(FLAIR, T2w) detection masks. Overall, across the configuration reported in Table 8, the mean Dice index for segmentation results is 61% (with 95% CI 0.56 to 0.66), which is satisfying considering the difficulty of the problem. An example of the segmentations obtained is presented in Figure 4.

Since the detection and segmentation phases can be done independently, we computed the performance of segmentation on the T2w, using FLAIR and ens.(FLAIR, T2w) detection masks. Despite T2w detection, segmentation using T2w did not fail. However, its results were still below FLAIR ones. On the T2w, pYU performance exhibits a dependence on the detection performance. Indeed, the pYU segmentation model failed to discriminate between tumoral voxels and healthy tissue ones, thus the segmentation results follow the detection performance. This is not the case for sYBBU, using ens.(FLAIR, T2w) detection masks, which have lower precision scores, and did not impact the segmentation model as much as the pYU. Looking at the Dice measurements, FLAIR and ens.(FLAIR, T2w) have similar performance whichever the bounding-boxes and the model used. However, FLAIR tends to have a higher precision while ens.(FLAIR, T2w) has a better recall. The choice between the two approaches should be made in regards to the application.

On the FLAIR, the Deepmedic network was outperformed by the detection model, and therefore obviously outperformed by the segmentation models that use the FLAIR mask. Deepmedic also failed to detect any tumour region on the same 9 cases excluded earlier.

## 5. Discussion

Our study proposes two detection-segmentation combination strategies that allowed us to obtain better results than the tested state-of-the-art networks (UNet and Deepmedic) on both BraTS’19, an openly available HGG and LGG dataset, and DIPG, a cohort of a rare paediatric tumour. Our strategies were able to segment the DIPG lesion while only training the models on the HGG cohort and without re-adapting the networks to the new tumour type. It was necessary to use this domain adaptation since we did not have access to enough annotated DIPG data nor a complete dataset to fine-tune each of the networks used.

Throughout this work, the FLAIR modality consistently appeared as the most important modality for any segmentation model, aiming at delineating globally the tumour lesion without distinguishing between its multiple compartments. It is therefore not surprising that our detection-segmentation algorithms prefer to rely on the FLAIR sequence. Moreover, the FLAIR modality has also been found as the most relevant for oncologists and features extracted from the FLAIR scans have shown the best results for survival analysis and tumour characterization for a range of tumours [45]. Specifically in DIPG, Castel et al. [3] identified differences in FLAIR index according to the type of histone mutated. Our segmentation, which is based on FLAIR imaging and produces a FLAIR-mostly derived delineation, produces regions of interest that appear to be relevant. Overall, having the FLAIR sequence for further imaging investigation on DIPG is a priority. In addition to that it appears that, even if the T2w did not perform as well as expected for the DIPG dataset, its presence always helped the proposed segmentation models.

Our proposals consist of procedures implicating multiple different and distinct models. Having different models, trained separately, has several advantages. The models had very different architectures, and therefore, could have different weaknesses and strengths, which can be complementary. In the DIPG case, even when the T2w detection failed, we were able to use the trained T2w BB-UNet model efficiently using the alternate FLAIR bounding-boxes. This possibility allowed us to circumvent the differences between glioblastoma and DIPG.

Our proposed approaches consist in combining multiple models, each model is relatively small. The inference time for each 3D example is around 5 s on an Nvidia Titan X, including 2.5 s for detection and 2.5 s for segmentation. Meanwhile, the training phase took roughly 4 h each. Comparatively, training Deepmedic took 3 days and had an inference time of 3 min per 3D example, on similar machine and software configurations.

Most recent segmentation efforts have focused on developing deeper and more complex models. While these solutions can be suitable for tumour lesions for which large curated and well-documented datasets, there is no indication that they can be easily adapted to small cohorts of rare tumours, such as DIPG, with missing data and heterogeneous quality. We found that Deepmedic, trained with four modalities (FLAIR, T2w, T1w, T1wgd), performed exceptionally well for HGG, with an average Dice of 0.9, but fails on DIPG with an average Dice of 0.3. In our proposition, the segmentation model is not fully dependent on the object detection performance, given bounding-boxes can be obtained from other input images. This allows us to use the best bounding-boxes assessed during a quality check. Our results are in line with the work of Isensee et al. [46], which found that recent efficient very complex and deep networks cannot necessarily be easily fine-tuned for rare oncological lesions segmentation problems with few training examples and, promoted the UNet architecture.

Our study presents several limitations. The ground-truth segmentations obtained on the DIPG are done on thick slices of 4 mm^3^, which negatively bias the results obtained even if it does not question the magnitude of these results. Furthermore, we have only one set of rare tumour data, further studies should investigate the robustness of the method using other rare tumours. Additionally, throughout this study, the used networks are considered black boxes. An ablation study could be made to investigate the limitation of the networks. We also did not investigate what the networks learnt and how they make the inference. Finally, we only focused on binary segmentation using either the FLAIR or T2w, further studies should investigate multi-compartment segmentation possibly using other modalities.

## 6. Conclusions

This paper addresses the problem of rare tumour types, for which no database can be built to train a deep neural segmentation network. Our work shows that state-of-the-art segmentation methods perform poorly when applied on test cohorts on which they were not trained. We propose to combine different simple models of detection and segmentation to allow us, not only to improve UNet performance but also to obtain satisfying results on a cohort that contained differences compared to the training dataset regarding, among others, patient age, image quality and tumour type.

Although all the sets presented in the paper present cerebral tumours, the differences between an adult brain (in the case of the HGGs and LGGs) and children brains (in the case of DIPG) give rise to challenges during inference. We think that using a set of a wider range of brain tumour types in children might help solve this issue. Additionally, the paper does not explore alternatives to the object detection framework YOLO. Work should be done to compare it to other algorithms, especially the ones dedicated to medical imaging and not only natural images. Lastly, other detection-segmentation strategies, such as the weak supervision paradigm, can be explored and compared to the proposed approaches.

We were able to obtain satisfying segmentation for the DIPG. These segmentations and performance will allow us to perform further clinical work to characterise this rare pathology using radiomics [47]. 

## Figures and Tables

**Table 1:** Dataset sizes of the different training and testing sets.

Dataset	HGG${}^{\mathrm{train}}$	HGG${}^{\mathrm{val}}$	TCGA-GBM${}^{\mathrm{test}}$	LGG${}^{\mathrm{test}}$	DIPG${}^{\mathrm{test}}$
number of patients	142	15	97	76	30 (71 sessions)
number of 2D images	9533	783			

**Table 2:** Detection results on TCGA-GBM${}^{test}$ with 97 test patients. Results present the mean ± standard deviation.

			With Morphological Transformation	
	Precision	Recall	Precision	Recall
FLAIR	0.577(±0.104)	0.899(±0.112)	0.599(±0.105)	0.927(±0.075)
T2w	0.569(±0.110)	0.880(±0.152)	0.593(±0.102)	0.905(±0.115)
ens.(FLAIR, T2w)	0.511(±0.103)	0.945(±0.069)	0.527(±0.105)	0.956(±0.059)

**Table 3:** Detection results on LGG${}^{test}$ with 76 test patients. Results present the mean ± standard deviation.

			With Morphological Transformation	
	Precision	Recall	Precision	Recall
FLAIR	0.577(±0.125)	0.849(±0.203)	0.611(±0.124)	0.873(±0.183)
T2w	0.581(±0.122)	0.856(±0.158)	0.610(±0.135)	0.883(±0.136)
ens.(FLAIR, T2w)	0.503(±0.121)	0.926(±0.121)	0.529(±0.123)	0.940(±0.094)

**Table 4:** Segmentation results on TCGA-GBM${}^{test}$ with 97 test patients. Unlike (BB-)UNet approaches, Deepmedic network [19] is trained on 3D volumes from the HGG${}^{train}$ dataset. Results present the mean ± standard deviation.

Architecture		Without Masking				With Masking		
		Precision	Recall	Dice		Precision	Recall	Dice
UNet	FLAIR	0.746±0.295	0.825±0.100	0.741±0.215	pYU *	0.902±0.128	0.813±0.103	0.845±0.089
	T2w	0.696±0.275	0.823±0.163	0.699±0.230		0.858±0.140	0.812±0.168	0.813±0.134
	ens.(FLAIR, T2w)	0.784±0.260	0.843±0.103	0.781±0.192		0.914±0.115	0.830±0.107	0.861±0.088
BB-UNet *	FLAIR	0.906±0.120	0.809±0.091	0.847±0.087	sYBBU *	0.921±0.102	0.807±0.093	0.854±0.079
	T2w	0.887±0.090	0.807±0.127	0.838±0.096		0.901±0.083	0.806±0.129	0.843±0.094
	ens.(FLAIR, T2w)	0.909±0.114	0.834±0.094	0.863±0.087		0.925±0.096	0.835±0.096	0.869±0.079
DeepMedic	FLAIR	0.913±0.110	0.774±0.175	0.820±0.149				

* Models using YOLO bounding-boxes.

**Table 5:** Comparison of segmentation performance when using the real bounding-boxes and predicted bounding-boxes for the FLAIR without post-processing. Results present the mean ± standard deviation.

	Precision	Recall	Dice
YOLO Bounding-Boxes	0.906±0.120	0.809±0.091	0.847±0.087
Real Bounding-Boxes	0.932±0.072	0.875±0.073	0.899±0.043

**Table 6:** Segmentation results on LGG${}^{test}$ with 76 test patients. Results present the mean ± standard deviation.

Architecture		Without Masking				With Masking		
		Precision	Recall	Dice		Precision	Recall	Dice
UNet	FLAIR	0.541±0.360	0.845±0.139	0.577±0.296	pYU *	0.863±0.155	0.768±0.172	0.792±0.142
	T2w	0.467±0.305	0.897±0.172	0.523±0.295		0.785±0.195	0.831±0.195	0.766±0.174
	ens.(FLAIR, T2w)	0.345±0.275	0.949±0.062	0.444±0.303		0.871±0.157	0.800±0.160	0.814±0.135
BB-UNet *	FLAIR	0.844±0.165	0.773±0.199	0.790±0.169	sYBBU *	0.878±0.144	0.772±0.201	0.804±0.165
	T2w	0.828±0.144	0.797±0.151	0.801±0.150		0.861±0.132	0.796±0.181	0.815±0.148
	ens.(FLAIR, T2w)	0.835±0.161	0.822±0182	0.815±0.153		0.871±0.141	0.820±0.183	0.831±0.152
DeepMedic	FLAIR	0.904±0.146	0.695±0.254	0.743±0.215				

* Models using YOLO bounding-boxes.

**Table 7:** Detection results on 62 sessions from the DIPG${}^{test}$ dataset excluding the 9 sessions where detection step failed to detect anything. Results present the mean ± standard deviation.

	With Morphological Transformation	
	**Precision**	**Recall**
FLAIR	0.606±0.185	0.664±0.237
T2w	0.471±0.285	0.363±0.334
ens.(FLAIR, T2w)	0.447±0.189	0.734±0.203

**Table 8:** Segmentation results on 62 sessions from DIPG, using FLAIR detection masks. Results present the mean ± standard deviation.

Architecture		Without Masking				With Masking		
		Precision	Recall	Dice		Precision	Recall	Dice
UNet	FLAIR	0.322±0.235	0.644±0.225	0.370±0.210	pYU *	0.753±0.206	0.571±0.243	0.611±0.215
	T2w	0.051±0.048	0.749±0.232	0.091±0.077		0.680±0.192	0.616±0.244	0.606±0.202
	ens.(FLAIR, T2w)	0.164±0.172	0.703±0.234	0.223±0.173		0.695±0.205	0.622±0.245	0.609±0.212
BB-UNet *	FLAIR	0.517±0.220	0.699±0.236	0.555±0.195	sYBBU *	0.733±0.194	0.598±0.244	0.622±0.206
	T2w	0.423±0.193	0.701±0.231	0.496±0.190		0.697±0.195	0.576±0.233	0.596±0.200
	ens.(FLAIR, T2w)	0.437±0.201	0.719±0.235	0.509±0.192		0.711±0.195	0.603±0.243	0.616±0.202
DeepMedic	FLAIR	0.624±0.225	00.614±0.259	0.558±0.240				

* Models using YOLO bounding-boxes.

## Data Availability

The data supporting the findings of this study are available within the article. DIPG data is not publicly available.

## Supplementary Materials

The following are available online at https://www.mdpi.com/article/10.3390/cancers13236113/s1, Figure S1: Details of the six different models that are trained for each modality. These 6 models will be used for inference either independantly, or serialized parallelized, Figure S2: The trained models are used for inference either independantly, or serialized parallelized. Finally results may be ensembled across the modality, Figure S3: Experimental design diagram, Table S1: YOLO parameters used for training, Table S2: Correlation study. Correlation values between detection precisions and final segmentation results obtained on the ensembled bounding-boxes, Table S3: Correlation study. Correlation values between detection recalls and final segmentation results obtained on the ensembled models, Figure S4: Mean precision-recall graphs of the different proposed segmentations on the LGG${}^{test}$ dataset. To focus on the most interesting region, we only plotted the precision-recall scores for thresholds between 0.1 and 0.9. From the left to the right, using the FLAIR, using the T2w, and ens.(FLAIR, T2w), Table S4: Detection results on 71 test cases from the DIPG set, Table S5: Segmentation results on 62 test sessions from the DIPG set, using ens.(FLAIR, T2w) detection masks.
