# Supplementary material for: Object Detection Improves Tumour Segmentation in MR Images of Rare Brain Tumours
Source: Cancers (Basel). 2021 Dec 4;13(23):6113. doi: 10.3390/cancers13236113 (PMC8657375; doi:10.3390/cancers13236113)
Supplement: Supplementary file 1 [file cancers-13-06113-s001.zip › cancers-1468888-supplementary.pdf]

Supplementary Materials: Object Detection Improves Tumour Segmentation in MR Images of Rare Brain Tumours

Hamza Chegraoui [1,*], Cathy Philippe [1], Volodia Dangouloff-Ros [2], Antoine Grigis[1], Raphael Calmon[2], Nathalie Boddaert[2], Frédérique Frouin[4], Jacques Grill[3] and Vincent Frouin[1,*]

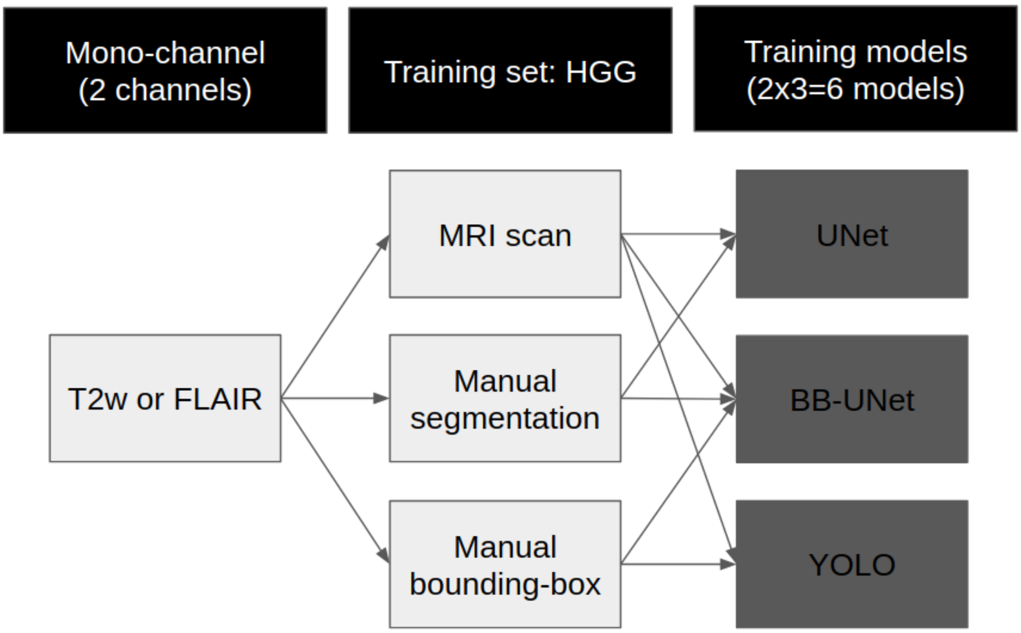

Figure S1. Details of the 6 different models that are trained for each modality. These 6 models will be used for inference either independantly, or serialized parallelized.

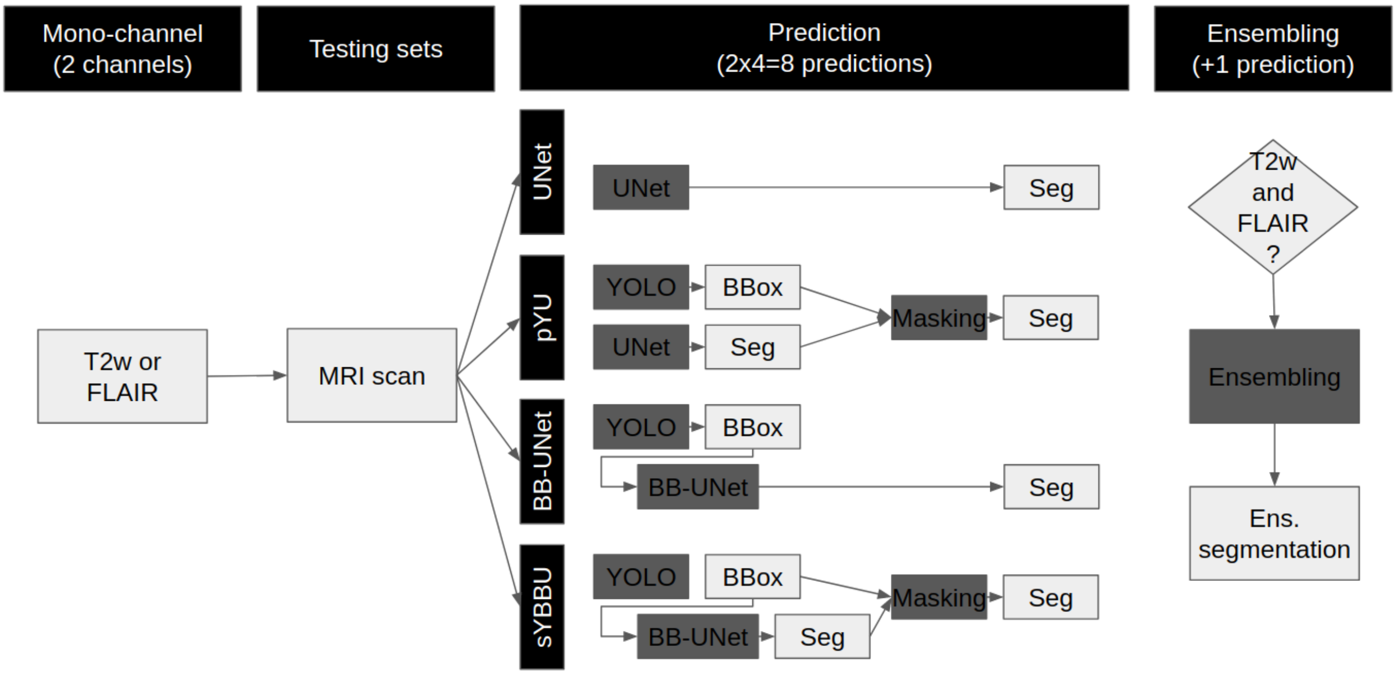

Figure S2. The trained models are used for inference either independantly, or serialized parallelized. Finally results may be ensembled accross the modality.

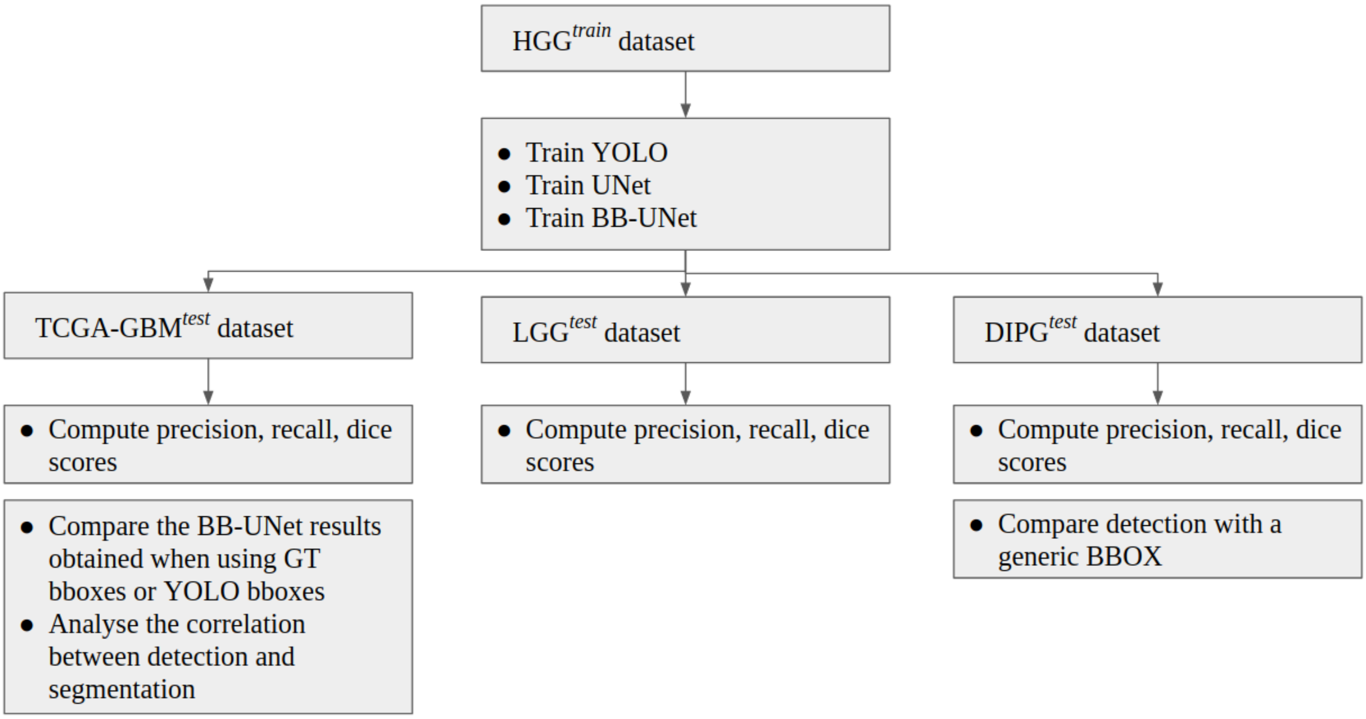

Figure S3. Experimental design diagram

Table S1. YOLO parameters used for training

		Parameters
Gradient	lr0	0.00320
	lrf	0.12000
	momentum	0.84300
Training	weight_decay	0.00036
	warmup_epochs	2.00000
	warmup_momentum	0.50000
	warmup_bias_lr	0.05000
Loss function	box	0.02960
	cls	0.24300
	cls_pw	0.63100
	obj	0.30100
	obj_pw	0.91100
	iou_t	0.20000
	anchor_t	2.91000
Augmentation	degrees	0.37300
	translate	0.24500
	scale	0.89800
	shear	0.60200
	perspective	0.00000
	flipud	0.00856
	fliplr	0.50000

Table S2. Correlation study. Correlation values between detection precisions and final segmentation results obtained on the ensembled bounding-boxes.

	Precision	Recall	Dice
UNet	0.400	-0.083	0.334
pYU	0.665	-0.073	0.422
BB-UNet*	0.615	-0.037	0.363
sYBBU	0.682	-0.033	0.357

Table S3. Correlation study. Correlation values between detection recalls and final segmentation results obtained on the ensembled models.

	Precision	Recall	Dice
UNet	0.167	0.665	0.319
pYU	0.000	0.762	0.615
BB-UNet*	0.189	0.791	0.684
sYBBU	0.081	0.804	0.716

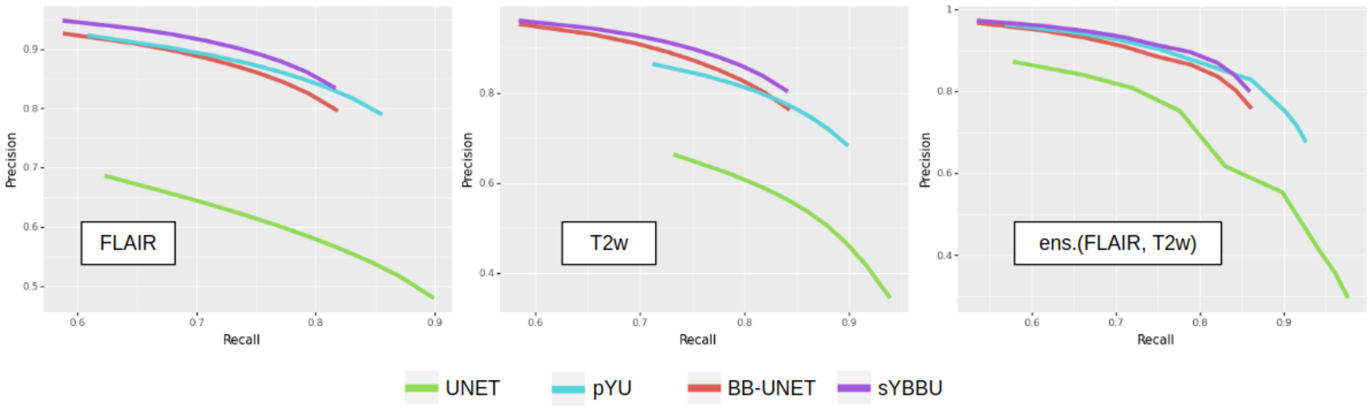

Figure S4. Mean precision-recall graphs of the different proposed segmentations on the LGG^{test} dataset. To focus on the most interesting region, we only plotted the precision-recall scores for thresholds between 0.1 and 0.9.From the left to the right, using the FLAIR, using the T2w, and ens.(FLAIR, T2w)

Table S4. Detection results on 71 test cases from the DIPG set.

	Precision	Recall
FLAIR	0.529 ± 0.265	0.580 ± 0.313
T2w	0.436 ± 0.299	0.342 ± 0.341
ens.(FLAIR, T2w)	0.395 ± 0.229	0.667 ± 0.305

Table S5. Segmentation results on 62 test sessions from the DIPG set, using ens.(FLAIR, T2w) detection masks.

		Precision	Recall	Dice
pYU	FLAIR	0.73 ± 0.20	0.59 ± 0.24	0.62 ± 0.21
	T2w	0.66 ± 0.19	0.63 ± 0.24	0.61 ± 0.19
	ens.(FLAIR, T2w)	0.67 ± 0.20	0.63 ± 0.24	0.61 ± 0.20
sYBBU	FLAIR	$.725 \pm .198$	$.609 \pm .205$	$.627 \pm .204$
	T2w	$.677 \pm .192$	$.596 \pm .203$	$.599 \pm .194$
	ens.(FLAIR, T2w)	$.688 \pm .192$	$.622 \pm .203$	$.618 \pm .197$
